# MiR-125a-3p and MiR-320b Differentially Expressed in Patients with Chronic Myeloid Leukemia Treated with Allogeneic Hematopoietic Stem Cell Transplantation and Imatinib Mesylate

**DOI:** 10.3390/ijms221910216

**Published:** 2021-09-23

**Authors:** Juliana R. B. Martins, Leonardo N. Moraes, Sarah S. Cury, Juliana Capannacci, Robson Francisco Carvalho, Célia Regina Nogueira, Newton Key Hokama, Paula O. M. Hokama

**Affiliations:** 1 Department of Internal Medicine, Botucatu Medical School, São Paulo State University (FMB-UNESP), Botucatu 18618-687, Brazil; juliana_ravelli@yahoo.com.br (J.R.B.M.); ju_capannacci@yahoo.com.br (J.C.); celia.nogueira@unesp.br (C.R.N.); newton.hokama@unesp.br (N.K.H.); 2 Department of Bioprocesses and Biotechnology, School of Agriculture, São Paulo State University (FCA-UNESP), Botucatu 18610-034, Brazil; leonardonmunesp@gmail.com; 3 Department of Structural and Functional Biology, Institute of Biosciences, São Paulo State University (IBB-UNESP), Botucatu 18618-970, Brazil; santiloni.cury@unesp.br (S.S.C.); robson.carvalho@unesp.br (R.F.C.)

**Keywords:** chronic myeloid leukemia, allogeneic hematopoietic stem cell transplantation, imatinib mesylate, miRNAs, miR-125a-3p, miR-320b

## Abstract

Chronic myeloid leukemia (CML), a hematopoietic neoplasm arising from the fusion of BCR (breakpoint cluster region) gene on chromosome 22 to the ABL (Abelson leukemia virus) gene on chromosome 9 (BCR-ABL1 oncogene), originates from a small population of leukemic stem cells with extensive capacity for self-renewal and an inflammatory microenvironment. Currently, CML treatment is based on tyrosine kinase inhibitors (TKIs). However, allogeneic hematopoietic stem cell transplantation (HSCT-allo) is currently the only effective treatment of CML. The difficulty of finding a compatible donor and high rates of morbidity and mortality limit transplantation treatment. Despite the safety and efficacy of TKIs, patients can develop resistance. Thus, microRNAs (miRNAs) play a prominent role as biomarkers and post-transcriptional regulators of gene expression. The aim of this study was to analyze the miRNA profile in CML patients who achieved cytogenetic remission after treatment with both HSCT-allo and TKI. Expression analyses of the 758 miRNAs were performed using reverse transcription quantitative polymerase chain reaction (RT-qPCR). Bioinformatics tools were used for data analysis. We detected miRNA profiles using their possible target genes and target pathways. MiR-125a-3p stood out among the downregulated miRNAs, showing an interaction network with 52 target genes. MiR-320b was the only upregulated miRNA, with an interaction network of 26 genes. The results are expected to aid future studies of miRNAs, residual leukemic cells, and prognosis in CML.

## 1. Introduction

Chronic myeloid leukemia (CML) was the first leukemia described in the literature, by Rudolf Virchow and John Hughes Bennett in 1845 [1]. Over the last 175 years, there have been a number of scientific discoveries involving CML and these have advanced the understanding and treatment of cancer. The understanding of the molecular pathogenesis of CML began with the discovery and correlation of t(9;22)(q34;q11) with the malignancy of the disease [2]. Followed by the identification of the chimeric BCR-ABL1 oncogene resulting from the translocation of the BCR (breakpoint cluster region) gene on chromosome 22 and ABL (Abelson leukemia virus) gene on chromosome 9 that encodes an elevated activity of oncoprotein tyrosine kinase [2].

CML is a clonal myeloproliferative neoplasm of hematopoietic stem cells with an incidence in adults of 1–2 cases per 100,000 inhabitants [3]. Three mechanisms are involved in CML oncogenesis, based on BCR-ABL1 activity: (a) altered cell adhesion to the bone marrow stroma and extracellular matrix, (b) constitutively active mitogenic signaling with reduced apoptosis of hematopoietic stem cells and progenitor cells, and (c) genetic instability leading to disease progression [4].

The identification of BCR-ABL1 tyrosine kinase as a therapeutic target has made possible the development of specific target drugs, administered orally, that molecularly control the tumor burden in CML. Imatinib mesylate (IM) was the first tyrosine kinase inhibitor (TKI) that was developed and approved for therapeutic use. IM acts as a competitive inhibitor of the ATP site on the BCR-ABL1 protein, blocking its tyrosine kinase activity and preventing the substrate from being phosphorylated. This block obstructs the transduction of signals necessary for cell proliferation [5]. Currently, new-generation TKIs are available for the treatment of CML; however, the only effective (curative) treatment of CML is allogeneic hematopoietic stem cell transplantation (HSCT-allo) [6].

HSCT-allo from a healthy allogeneic donor has been shown to consistently eradicate leukemic stem cells in most patients. The successful outcome of this treatment is related to the effectiveness of the leukemia graft reaction (LGR) orchestrated by allogeneic T cells [7].

In the last two decades, the advent of TKIs has changed the indication of HSCT-allo from an early and curative intervention to a salvage treatment, recommended for patients resistant to ITKs or in the advanced stages of the disease [6,8].

Given the evidence of therapeutic resistance to ITKs, recent advances in gene expression technology mechanisms have shown that microRNAs (miRNAs) have great potential both as therapeutic targets and as prognostic markers in different types of cancer [9,10,11].

MiRNAs are small non-protein coding RNAs that act as post-transcriptional regulators of gene expression [12]. Recently, several studies have demonstrated the potential of miRNAs as regulators of physiological and pathological processes [13]. MiRNAs play an important role in T cell development and regulation, as well as in immune reconstitution after HSCT-allo [14]. Furthermore, miRNAs have been shown to play a crucial role in neoplasm pathogenesis, promoting changes such as cell proliferation, angiogenesis, tumor growth, and metastasis [15,16].

From the first descriptions, in 2002, of the relationship between miRNA and cancer, it was observed that cancer patients exhibit dysregulated expression levels of miRNAs, both positively and negatively [17]. Consequently, each type of cancer has a miRNA expression profile relative to its condition [18]. Furthermore, deregulated miRNAs may have functional oncogenic or tumor suppressor roles [19]. Examples of these mechanisms are miR-30e and miR-203 that act as tumor suppressors, downregulating BCR-ABL1 expression in CML [20,21]. Meanwhile, miR-486 can elevate imatinib resistance by targeting FOXO1 and PTEN in CD34 positive CML cells [22].

The aim of this study was to identify a miRNA profile, target genes, and target pathways in CML-chronic phase patient groups treated with HCST-allo and imatinib mesylate, both in cytogenetic remission and over 12 months of treatment. Expression analyses of the 758 miRNAs were performed using RT-qPCR. Bioinformatics tools were used for data analysis. We detected miRNA profiles using their possible target genes and target pathways. MiR-125a-3p stood out among the downregulated miRNAs, showing an interaction network with 52 target genes. MiR-320b was the only upregulated miRNA, with an interaction network of 26 genes.

## 2. Results

### 2.1. Differentially Expressed MiRNAs

Expression data for the miRNAs obtained in this study are available from the NCBI GEO database (accession number GSE 164549). A total of 758 miRNAs were analyzed using RT-qPCR array in peripheral blood samples from 14 patients with CML-chronic phase treated with imatinib mesylate and 14 patients treated with HSCT-allo, both groups in cytogenetic remission. According to the cut-off criteria (fold change < 0.5, fold change > 2.0), 43 differentially expressed miRNAs were identified from this microarray dataset. Of these miRNAs, one (2.3%) was upregulated and 42 (97.7%) were downregulated (Table 1).

### 2.2. MiRNAs Target Genes

The 43 differentially expressed miRNAs were analyzed using the miRWalk program (http://zmf.umm.uni-heidelberg.de/apps/zmf/mirwalk2/index.html, accessed on 27 August 2021) to identify possible miRNA target genes. Through this analysis we identified 1171 genes. These genes were verified and identified as differentially expressed using microarray data available in the Gene Expression Omnibus (http://www.ncbi.nlm.nih.gov/geo, accessed on 23 September 2020), accession number GSE 43225, in a study conducted on CML patients. Among the genes we found in our study, 95% were also identified among the genes in the microarray data from the Gene Expression Omnibus.

**Figure 1:**
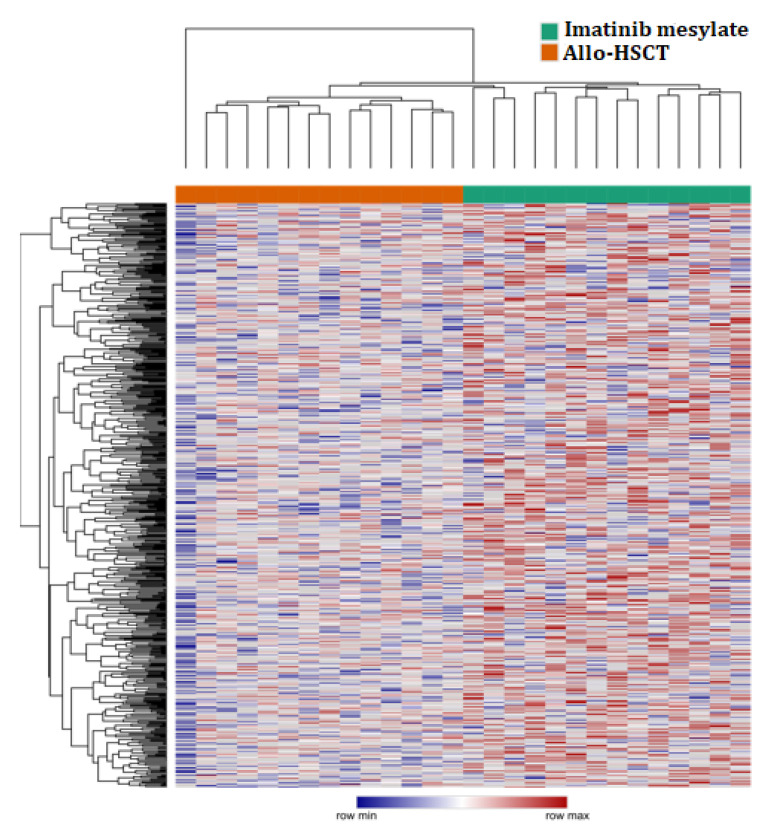
Heatmap of the normalized expression values of microRNAs in allo-HSCT and imatinib mesylate groups.

MiRNA expression in all patients treated with imatinib mesylate and HSCT-allo using heatmap is shown in Figure 1. Heatmap Clusterization was performed using Euclidean distance. Heatmap was generated and analyzed using Morpheus (https://software.broadinstitute.org/morpheus, accessed on 27 August 2021).

### 2.3. Protein–Protein Interaction Network

**Figure 2:**
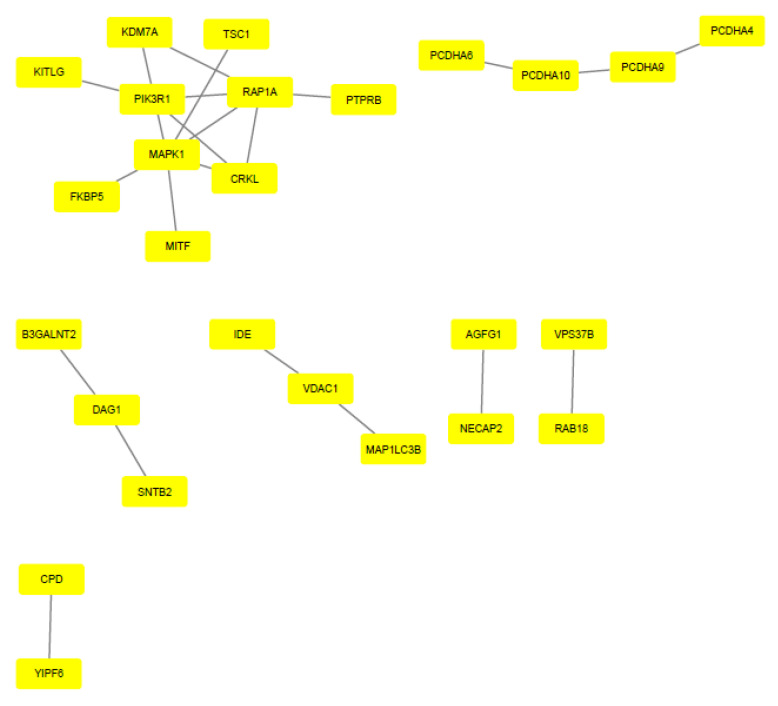
Protein–protein interaction of upregulated miRNA target genes. Gene interaction was performed using STRING and Cytoscape tools. Genes are represented by yellow rectangles and lines represent protein–protein associations.

**Figure 3:**
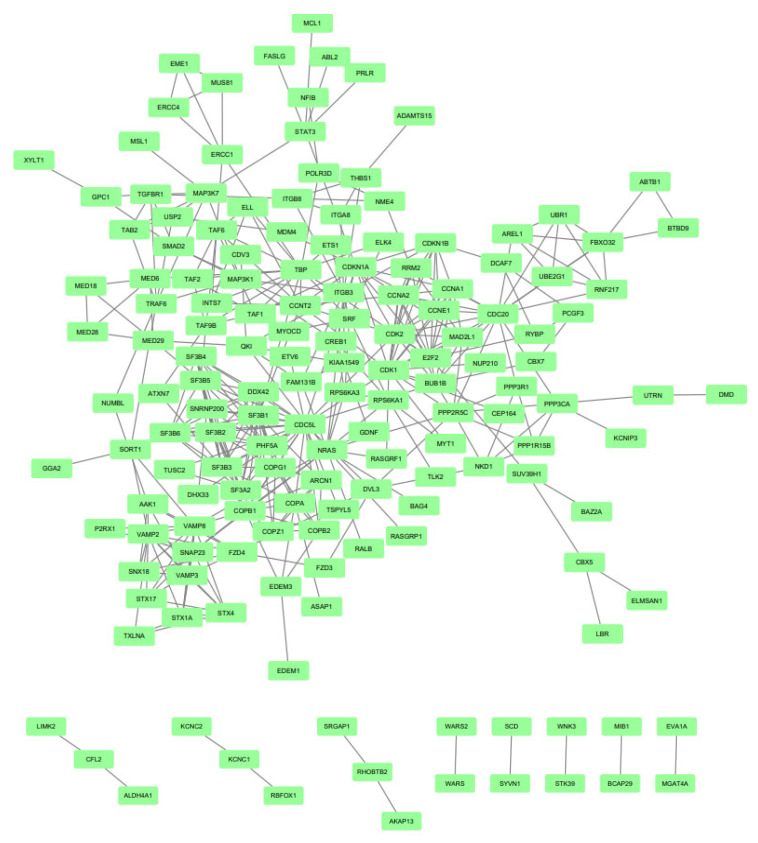
Protein–protein interaction of downregulated miRNAs target genes. Gene interaction was performed using STRING and Cytoscape tools. Genes are represented by green rectangles and lines represent protein–protein associations.

The EnrichR tool (Mount Sinai Innovation Partners, New York, NY, USA) http://amp.pharm.mssm.edu/Enrichr/, accessed on 24 September 2020) was used to visualize the main pathways related to the target genes of the miRNAs. The Reactome program provided a biological interpretation and provided models to visualize the results. STRING (version 11.0; (String Consortium, Zurich, Switzerland), https://string-db.org/, accessed on 27 September 2020) was performed on the target genes of upregulated and downregulated miRNAs, generating data visualized through pictures created by Cytoscape software (version 3.8.0; Cytoscape Consortium, Bethesda, MA, USA), http://www.cytoscape.org, accessed on 28 September 2020) (Figure 2 and Figure 3).

### 2.4. Gene Ontology

Based on the network results, the genes that showed the greatest interaction with each other were selected, according to the figures generated by Cytoscape. For these genes, which were considered as the main genes, an analysis was performed using Gene Ontology (http://geneontology.org/, accessed on 24 September 2020) to identify the pathways involved with these genes. The main terms found are listed in Table 2 and Table 3.

**Figure 4:**
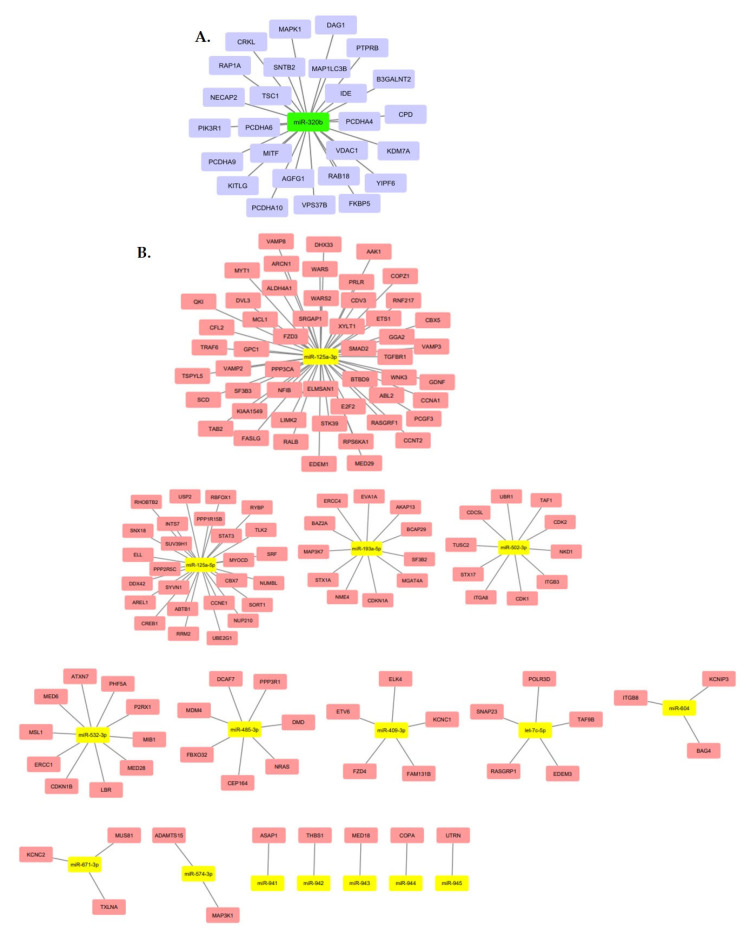
Main upregulated miRNA target genes (**A**). Main downregulated miRNA target genes (**B**).

**Figure 5:**
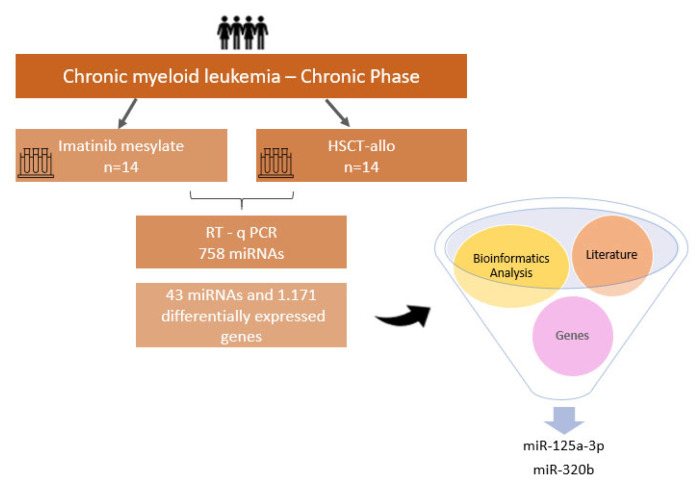
Summary of methodology steps and main results.

From the profile of 43 identified miRNAs, we highlighted two miRNAs: miR-320b and miR-125a-3p. MiR-320b was the only upregulated miRNA found, and among those with low expression, miR-125a-3p was the one with the highest gene interaction, standing out among the results. MiR-125a-3p showed an interaction network with 52 target genes, and miR-320b showed an interaction network with 26 genes. The main target genes related to miR-320b and miR-125a-3p are shown in Figure 4. A summary of the methodology steps and the main results are presented in Figure 5.

## 3. Discussion

Chronic myeloid leukemia (CML) is a hematopoietic neoplasm that arises from BCR-ABL1 translocation, originating from a small population of leukemic stem cells with extensive self-renewal capacity and an inflammatory microenvironment. Currently, CML treatment is based on tyrosine kinase inhibitors and target-specific drugs that are safe and effective. However, HSCT-allo was the first treatment in CML patients to eradicate the Philadelphia chromosome. In this process, after high doses of chemotherapy, transplanted hematopoietic stem cells can reestablish the recipient’s hematopoiesis [23].

Currently, HSCT-allo is the only treatment available that promotes a CML cure; however, morbidity and mortality rates related to this treatment are high, in addition to the difficulty of finding a donor, restricting its applicability [24,25,26,27]. Therefore, after the advent of ITK, HSCT-allo began to be used for CML treatment only in patients who developed therapeutic resistance [28].

The first ITK approved for use was imatinib mesylate [29,30], a potent TKI [31,32,33]. However, 10–15% of patients in the chronic phase of CML do not show favorable changes with the use of imatinib mesylate due to resistance or intolerance to the treatment [34,35]. Despite recent advances in CML treatment, these challenges remain and prevent the development of effective therapeutic strategies [36]. With new research of miRNAs, possible new treatment mechanisms are being discovered.

In recent years, many studies have focused on the significant role of miRNAs in physiological and pathological processes [37]. These small RNAs can be identified in the peripheral blood and are used as biomarkers for several diseases [38,39]. Evidence indicates that miRNAs are directly involved in myeloid development and leukemogenesis [40].

In addition to miRNA-based studies, bioinformatics has also contributed to the understanding of processes linked to the development and progression of CML, along with the discovery of possible disease markers. Recent studies have shown increasing evidence of the interaction between genes and proteins, leading to investigations into molecular cancer mechanisms. In this context, bioinformatics is essential for integrating biological systems and computational science, helping to better understand the diagnosis processes, therapy, and prognosis of cancer and other diseases [41].

In our study, we compared two CML patient groups. One group was treated with HSCT-allo, and the other group was treated with imatinib mesylate. The clinical follow-up of transplant patients averaged between 9.6 years and 4.5 years for those treated with imatinib mesylate. In this follow-up period, we observed that five of the 14 (35.7%) patients treated with ITK achieved a deep molecular response (MR^4log^, MR^4.5Log^ or undetectable) throughout the treatment and another five patients (35.7%) achieved a major molecular response (MR^3Log^). On the other hand, nine patients (64.3%) treated with HSCT-allo relapsed and five (35.7%) achieved major molecular response (MR^3Log^). By analyzing the miRNAs of these two patient groups using bioinformatics tools, it was possible to obtain a profile consisting of 43 differentially expressed miRNAs, one (2.3%) upregulated and 42 (97.7%) downregulated (Table 1).

### 3.1. Upregulated MiR-320b and Differentially Expressed CRKL

The treatment of cancer patients has evolved considerably; however, cell regeneration and the ability of stem cells to self-renew are significant obstacles in this process, as they can lead to tumor recurrence, metastasis, and drug resistance. MiRNAs play a fundamental role in this process, as they are unregulated in several malignant diseases and are important regulators of stem cells and cell reprogramming [42].

MiR-320, the only upregulated miRNA found in our study, was studied by Gao et al. [43], who showed that miR-320, secreted by leukemic cells, is directly related to increased cell proliferation and that leukemic cell exosomes can transport miRNAs to stromal cells, leading to reprogramming of cell niche functions.

Regarding miR-320b target genes, our data pointed to the low expression of CRKL in both groups of patients evaluated. The CRKL family is composed of five members: v-CRK, CRKI, CRKII, CRKIII, and CRL-like protein (CRKL) [44]. CRK proteins are phosphorylation substrates for the BCR-ABL1 fusion oncogene found in over 95% of CML cases. CRKL is a tyrosine-phosphorylated protein found in neutrophils from patients with CML [44]. We believe that the overexpression of miR-320b and the low expression of CRKL found in this study is a consequence of the absence of a deep molecular response in the patient groups evaluated.

### 3.2. MiR-125a-3p, MiR-485-3p, MiR-409-3p and MiR-574-3p Downregulated

Of the 43 differentially expressed miRNAs identified in our study, 42 (97.7%) were downregulated. Among the 42 downregulated miRNAs, we will highlight miR-125a-3p, miR-485-3p, miR-409-3p, and miR-574-3p, due to their relevance in the literature. This set of miRNAs was described by Xiong and collaborators as differentially expressed in the K562 cell line, BCR-ABL1 positive [45].

Low expression of miR-125a has been detected in bone marrow samples from patients with acute myeloid leukemia (AML) compared to the control group (healthy bone marrow donors) [38]. Previous studies have demonstrated that miR-125a plays a role in cell cycle regulation, proliferation, and apoptosis in AML [46]. In acute promyelocytic leukemia, miR-125b was found differentially expressed, promoting the proliferation of leukemic cells, and inhibiting cellular apoptosis by regulating the expression of the tumor suppressor BCL2-antagonista/killer1 (Bak1) [47].

MiR-485 has been described as poorly expressed in AML HL60 cell lines when compared to normal peripheral blood cells [48]. In a study by Li et al., miR-485-3p was one of 33 miRNAs differentially expressed in a CML patient group. These findings contribute to the understanding of the disease pathogenesis [49].

MiR-409-3p is also a miRNA described in the literature as having reduced expression in CpG-rich methylated CML cell lines [50,51]. This low expression of miRNAs has been associated with a high relapse risk in AML [52,53]. Low expression of miR-574-3p was also observed in CML patients when compared to samples from healthy controls, indicating the involvement of this miRNA in the development and progression of the disease [54].

These findings are expected to contribute to the understanding of disease pathogenesis and persistence of residual BCR-ABL1 cells observed in our patient groups after different therapeutic strategies.

### 3.3. MAPK and NRAS

After analyzing the target genes, we used Gene Ontology to study the enrichment of the pathways of these genes and to observe the main pathways related to these genes. Some pathways were found to be fundamental to CML development, such as regulation of protein kinase activity, processes related to cell division, and mitogen-activated-protein kinase (MAPK) activity.

The MAPK pathway is an important signaling cascade found in several types of cancer [55] and plays a central role in CML, as it is necessary for the transcription of genes involved in cell proliferation [56,57]. In our results, we found a high expression of MAP3K1 and MAP3K7 genes. These genes are members of the MAPK family [58,59]. MAP3K1 is involved in the survival and migration of tumor cells [60], and MAP3K7 is an important regulator of cell pathways associated with cell proliferation in cancer [59]. NRAS was also found to be highly expressed in our study. The NRA family is composed of three genes associated with carcinogenesis: HRAS, KRAS, and NRAS [61]. Mutations in NRAS are often found in myeloid disorders such as CML [62]. MAPK and NRAS were found to be highly expressed in our study, suggesting residual tumor activity.

In the present study, we explored through bioinformatics tools the profile of miRNAs, their target genes, and related pathways in CML patients undergoing treatment. Protein–protein interaction networks were built to relate miRNAs found with their target genes, and it was possible to obtain data that corroborate the current literature. As this is an exploratory study with real-world data and scarce samples, it was not possible to validate the results through a second round of RT-qPCR. However, we believe that this study contributes an innovative approach and useful results to the fields of oncology and bioinformatics. We stress the need for expanded studies in order to confirm the miRNA profiles discussed in this work as markers of residual disease.

## 4. Materials and Methods

### 4.1. Patients and Samples

The study was approved by the Hospital Amaral Carvalho Ethics Committee (CEPHAC-2.917.389). Patients who were followed up at Hospital Amaral Carvalho signed a form attesting to free and informed consent (the FICF form) to participate in the study. Peripheral blood samples from 28 patients with CML-chronic phase in cytogenetic remission (Philadelphia chromosome-negative) were included in the study. The patients were divided into two groups: 14 patients undergoing HSCT-allo and 14 patients treated with imatinib mesylate. A pool of leucocytes from 14 healthy blood donors was considered as the control group. The miRNA profile was determined by comparing the control group and each patients’ group to determine which miRNAs were upregulated or downregulated. The patients’ clinical data are presented in Table 4. All transplant patients received BuCy-2 as a conditioning regimen and cyclosporine and methotrexate as graft-versus-host disease prophylaxis [63,64].

### 4.2. RNA Extraction and Purification

Total RNA was isolated from buffy coat of fresh peripheral blood using the QIAamp^®^ RNA Blood Mini Kit (QIAGEN, Hilden, Germany) according to the manufacturer’s protocol. The total RNA amount was determined by the ratios A 260 nm/A 280 nm and A 260 nm/A 230 nm (acceptable when both ratios were greater than 1.8). RNA integrity was ensured by obtaining an RNA integrity number (RIN > 8) using the Agilent 2100 Bioanalyzer (Agilent Technologies, Waldbronn, Germany) [65,66].

### 4.3. Expression MiRNAs Profile and Reference Genes

The reverse transcription reaction was performed using the Taqman MicroRNA Reverse Transcription Kit in combination with Megaplex RT Primer Human Pool Set A and B (Thermo Fisher Scientific, Waltham, MA, USA). This reaction transcribed 758 miRNAs and three positive endogenous controls (U6 snRNA, RNU44, and RNU48) and one negative control [65,66].

The TaqMan^®^ MGB strategy was used for quantitative real-time PCR (q-PCR), which is based on the specific annealing of each probe with its complementary sequence. For miRNA amplification, the Taqman^®^ Low Density Array Human MicroRNA Arrays v2.0 (A and B) kit (ABIV^®^, Life Technologies, Carlsbad, CA, USA) was used according to the manufacturer´s instructions. Analyses were performed on the ViiA7 platform (ABIV^®^) for a total of 758 miRNAs per patient [65,66].

### 4.4. Bioinformatics Analysis

Bioinformatic analysis programs were selected based on the options described by Reimand et al. [67]. Expression Suite Software Version 1.1 was used to identify differentially expressed miRNAs. MiRWalk 2.0 was used to study possible target genes of differentially expressed miRNAs, which includes target prediction data generated by different algorithms (including the algorithm itself) [68]. The following algorithms were selected: miRWalk, miRDB, Micro t4, miRanda, RNAhybrid, and Targetscan. Only targets predicted by at least three of the selected algorithms were accepted. The predicted targets previously identified as differentially expressed were then verified using microarray data available from the Gene Expression Omnibus (accession number GSE 43225). Microarray data were analyzed using GEO2R script (http://www.ncbi.nlm.nih.gov/geo/geo2r/, 23 September 2020). Differentially expressed genes were considered when they showed a fold change of at least 1.5. Gene Ontology was used to search for enriched terms among differentially expressed genes using Bonferroni´s correction and accepting only terms with *p* ≤ 0.05. Differentially expressed genes related to upregulated and downregulated miRNAs were analyzed according to EnrichR for enrichment analysis. The Reactome was used for data analysis, and the evaluation of protein–protein interaction network (PPI), based on a list of genes, was performed using the online tool STRING version 11.0. Experiments, databases, co-expression, neighborhoods, and co-occurrence were considered. The minimum interaction score was 0.700. Cytoscape software version 3.8.0 was used to view the results.

### 4.5. Statistic

A comparison analysis of Ct was used to quantify miRNA expression. Differences were statistically assessed using Student’s *t*-test. Statistical significance was set at *p* < 0.05.

## 5. Conclusions

It was possible to determine the miRNA profiles of patients treated with HSCT-allo and imatinib mesylate, as well as their target genes and pathways. The results of our study are expected to contribute to further research in the field of CML prognosis, in particular, the identifying of new biomarkers for this disease. Bioinformatics analyzes predict, in silico, interactions that require biological validation before clinical application.

## Figures and Tables

**Table 1:** Differentially expressed MiRNAS.

miR Name	miRBase ID	FC	*p*-Value
hsa-miR-20b	MIMAT0001413	0.128	0.000
hsa-miR-502-3p	MIMAT0004775	0.135	0.000
hsa-miR-127	MIMAT0000446	0.143	0.000
hsa-miR-485-3p	MIMAT0002176	0.148	0.000
hsa-miR-125a-3p	MIMAT0004602	0.174	0.001
hsa-miR-128a	MIMAT0000424	0.174	0.001
hsa-miR-92a	MIMAT0000092	0.188	0.000
hsa-miR-411	MIMAT0003329	0.189	0.034
hsa-miR-532-3p	MIMAT0004780	0.200	0.000
mmu-miR-140	MIMAT0000151	0.205	0.015
hsa-miR-218	MIMAT0000275	0.212	0.036
hsa-miR-125a-5p	MIMAT0000443	0.217	0.001
hsa-miR-30c	MIMAT0000244	0.225	0.003
hsa-miR-23a	MIMAT0000078	0.234	0.003
hsa-miR-598	MIMAT0003266	0.234	0.011
hsa-miR-362	MIMAT0000705	0.288	0.013
hsa-miR-616	MIMAT0004805	0.351	0.017
hsa-miR-331	MIMAT0000760	0.422	0.023
hsa-miR-193a-5p	MIMAT0004614	0.072	0.000
hsa-miR-574-3p	MIMAT0003239	0.089	0.000
hsa-miR-454	MIMAT0003885	0.136	0.001
hsa-miR-323-3p	MIMAT0000755	0.137	0.000
hsa-miR-671-3p	MIMAT0004819	0.153	0.000
hsa-let-7c	MIMAT0000064	0.173	0.000
hsa-miR-30b	MIMAT0000420	0.188	0.004
hsa-miR-532	MIMAT0002888	0.207	0.002
hsa-miR-146a	MIMAT0000449	0.233	0.002
hsa-miR-132	MIMAT0000426	0.293	0.004
mmu-miR-491	MIMAT0003486	0.293	0.005
hsa-miR-126	MIMAT0000445	0.328	0.046
hsa-miR-708	MIMAT0004926	0.336	0.017
hsa-miR-191	MIMAT0000440	0.380	0.009
hsa-miR-543	MIMAT0004954	0.141	0.001
hsa-miR-604	MIMAT0003272	0.232	0.011
hsa-miR-548J	MIMAT0005875	0.289	0.028
hsa-miR-409-3p	MIMAT0001639	0.142	0.001
hsa-miR-550	MIMAT0004800	0.190	0.000
hsa-miR-151-3p	MIMAT0000757	0.268	0.013
hsa-miR-941	MIMAT0004984	0.292	0.026
hsa-miR-1285	MIMAT0005876	0.357	0.026
hsa-miR-378	MIMAT0000731	0.387	0.018
hsa-miR-589	MIMAT0003256	0.402	0.036
hsa-miR-320b	MIMAT0005792	2.462	0.021

**Table 2:** Enriched terms generated by Gene Ontology for the main target genes of downregulated miRNAs.

Pathway ID	Pathway Description	*p*-Value	FDR
GO: 0090304	Nucleic acid metabolic process	2.34E-06	3.72E-02
GO: 0006139	Nucleobase-containing compound metabolic process	1.28E-05	1.01E-01
GO: 0072422	Signal transduction involved in DNA damage checkpoint	1.64E-05	8.69E-02
GO: 0072401	Signal transduction involved in DNA integrity checkpoint	1.64E-05	6.51E-02
GO: 0072395	Signal transduction involved in cell cycle checkpoint	1.71E-05	5.42E-02
GO: 0051329	Mitotic interphase	2.06E-05	5.46E-02
GO: 0051325	Interphase	2.06E-05	4.68E-02
GO: 0046483	Heterocycle metabolic process	2.25E-05	4.46E-02
GO: 0006725	Cellular aromatic compound metabolic process	2.58E-05	4.56E-02
GO: 0016070	RNA metabolic process	3.62E-05	5.75E-02
GO: 0000079	Regulation of cyclin-dependent protein serine/threonine kinase activity	3.97E-05	5.73E-02
GO: 0000398	mRNA splicing, via spliceosome	4.15E-05	5.48E-02
GO: 0000377	RNA splicing, via transesterification reactions with bulged adenosine	4.15E-05	5.06E-02
GO: 0000375	RNA splicing, via transesterification reactions	4.31E-05	4.88E-02
GO: 1904029	Regulation of cyclin-dependent protein kinase activity	4.45E-05	4.71E02
GO: 0042770	Signal transduction in response to DNA damage	4.71E-05	4.67E-02
GO: 1901360	Organic cyclic compound metabolic process	4.79E-05	4.47E-02
GO: 0072331	Signal transduction by p53 class mediator	7.11E-05	6.27E-02
GO: 0034641	Cellular nitrogen compound metabolic process	7.56E-05	6.31E-02
GO: 0000082	G1/S transition of mitotic cell cycle	8.56E-05	6.79E-02

Top 20 GO terms with a lower *p*-value in Biological Process. GO, Gene Ontology; FDR, false discovery rate.

**Table 3:** Enriched terms generated by Gene Ontology for the main target genes of upregulated miRNAs.

Pathway ID	Pathway Description	*p*-Value	FDR
GO: 0000186	Activation of MAPK activity	2.25E-07	3.58E-03
GO: 1903827	Regulation of cellular protein localization	2.74E-06	2.17E-02
GO: 1901699	Cellular response to nitrogen compound	4.93E-06	2.61E-02
GO: 0050952	T cell receptor signaling pathway	6.78E-06	2.69E-02
GO: 0050778	Positive regulation of immune response	7.60E-06	2.41E-02
GO: 1905475	Regulation of protein localization to membrane	8.82E-06	2.33E-02
GO: 0060341	Regulation of cellular localization	1.34E-05	3.03E-02
GO: 0043549	Regulation of kinase activity	1.80E-05	3.57E-02
GO: 0032880	Regulation of protein localization	2.19E-05	3.85E-02
GO: 1902533	Positive regulation of intracellular signal transduction	2.74E-05	4.35E-02
GO: 0051338	Regulation of transferase activity	2.90E-05	4.18E-02
GO: 0050851	Antigen receptor-mediated signaling pathway	3.00E-05	3.97E-02
GO: 0002684	Positive regulation of immune system process	3.16E-05	3.86E-02
GO: 1901701	Cellular response to oxygen-containing compound	3.38E-05	3.83E-02
GO: 1901698	Response to nitrogen compound	3.57E-05	3.78E-02
GO: 0032147	Activation of protein kinase activity	3.83E-05	3.80E-02
GO: 0046326	Positive regulation of glucose import	3.94E-05	3.68E-02
GO: 0050776	Regulation of immune response	4.26E-05	3.76E-02
GO: 0071495	Cellular response to endogenous stimulus	4.44E-05	3.71E-02
GO: 0042307	Positive regulation of protein import into nucleus	4.53E-05	3.60E-02

Top 20 GO terms with a lower *p*-value in Biological Process. GO, Gene Ontology; FDR, false discovery rate.

**Table 4:** Patients’ clinical characteristics.

Clinical Characteristics	HSCT-allo n = 14	Imatinib n = 14
Age mean at miRNA screening, years	40	50
Gender:		
Female	05	06
Male	09	08
BCR-ABL1 at miRNA screening, media (%)	0.37	1.15
BCR-ABL1 Breakpoint		
b2a2	06	04
b3a2	08	10
ABL Domain Mutation		
Presence	00	01 (M244V)
Absence	07	05
Not available	07	08
Follow up		
Time, media, years	9.6	4.5
BCR-ABL1 undetectable	00	05
MMR (BCR-ABL1 ≤ 0.1%)	05	05
Relapsed	09	02
Lost follow up	00	02

MMR: major molecular response.

## Data Availability

All datasets generated for this study are included in the article.
